# Syringin exerts anti‐inflammatory and antioxidant effects by regulating SIRT1 signaling in rat and cell models of acute myocardial infarction

**DOI:** 10.1002/iid3.775

**Published:** 2023-02-24

**Authors:** Di Zhao, Ketong Liu, Jian Wang, Haifeng Shao

**Affiliations:** ^1^ Department of Cardiology I The Third Affiliated Hospital of Qiqihar Medical University Qiqihar Heilongjiang China; ^2^ Department of Cardiology The Fifth Affiliated Hospital (Zhuhai) of Zunyi Medical University Zhuhai Guangdong China

**Keywords:** inflammation, myocardial ischemia/reperfusion, oxidative stress, SIRT1, syringing

## Abstract

**Introduction:**

This study aimed to investigate the role of syringin in improving heart function during myocardial ischemia/reperfusion (I/R) and to determine whether the sirtuin 1/peroxisome proliferator‐activated receptor gamma coactivator 1 alpha (SIRT1/PGC‐1α) pathway contributes to this cardioprotective effect in vivo and in vitro.

**Methods:**

H9c2 cells were incubated with H_2_O_2_ for 12 h. The effect of syringin was assessed by measuring cell viability; the apoptotic rate; Keap1/NRF2/HO‐1 activation; and the levels of proinflammatory cytokines, oxidative products, and antioxidative enzymes. In addition, SIRT1 was silenced via short hairpin RNA (shRNA)‐SIRT1 transfection to evaluate its involvement in syringin‐mediated protection. Syringin rescued cells from H_2_O_2_‐induced reductions in viability, antioxidative enzyme levels, and NRF2/HO‐1 activation; likewise, syringin inhibited apoptosis, inflammation, and oxidative stress. We also created a rat model of I/R by ligating the left anterior descending coronary artery for 30 min, followed by reperfusion for 12 min. Syringin was then intraperitoneally injected, and the effect on infarct size and cardiac function was examined after 7 days. NRF2/HO‐1 activity and the levels of myocardial proinflammatory cytokines, oxidative products, and antioxidative enzymes were measured.

**Results:**

In comparison to the untreated I/R group, the syringin treatment group exhibited improved cardiac function and reduced cardiac lesion and infarct size. Syringin administration also markedly reduced the levels of proinflammatory cytokines and reactive oxygen species and promoted antioxidative enzyme expression and NRF2/HO‐1 pathway activation.

**Conclusions:**

Syringin may serve a protective role in animal and cell models of I/R by improving cardiac function, inhibiting the inflammatory response, and activating the antioxidative response.

## Graphical abstract

The present study revealed that the sirtuin 1 contributes to syringin‐mediated protection against myocardial ischemia/reperfusion in rats. Our findings provide both novel insight into the mechanism of cardioprotection conferred by syringin, as well as further evidence for its potential value in the development of new treatments for myocardial infarction.

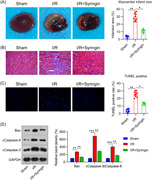

## 1 INTRODUCTION

Acute myocardial infarction (MI), the most common factor influencing cardiovascular disease occurrence and mortality worldwide, is caused by coronary occlusion.^1^ Acute MI results in reduced cardiovascular output and circulatory arrest, and cardiac surgery is often required.^2^ Yet despite its prevalence, the complex biological mechanisms governing MI damage remain poorly understood.

Syringin, a bioactive compound, is isolated from *Acanthopanax senticosus*.^3^ Previous studies have shown that syringin has multiple pharmacological functions, including protection of the liver and kidneys and reduction of inflammation and oxidative stress (OS).^4^ Zhou et al.^5^ found that in mice with radiation‐induced brain injury, syringin levels are increased in response to the blocked flow of blood to the brain. Syringin also attenuates neuronal cytotoxicity and apoptosis,^6^ and several studies have shown that it protects against lipopolysaccharide‐induced acute lung injury.^7^ Likewise, syringin decreases inflammation and cerebral damage in rats with cerebral ischemia/reperfusion (I/R) injury. Mechanistically, the neuroprotective effect of syringin may involve TLR4 suppression.^8^ However, both the role of syringin in myocardial I/R damage and the mechanism of its cardioprotective function remain to be clarified.

Sirtuin 1 (SIRT1), a nicotinamide adenine dinucleotide‐dependent histone deacetylase, plays a role in cell survival, longevity, regulation of metabolism, and differentiation,^9^ as well as in cell cycle control, gene silencing, cell death, and energy homeostasis.^10^, ^11^, ^12^ SIRT1 is associated with nuclear steroid receptor coactivators, such as peroxisome proliferator‐activated receptor gamma coactivator 1 alpha (PGC‐1α), PPARγ, and p300, and is involved in liver metabolism, fat storage and production, and myocyte differentiation.^13^, ^14^ Conversely, SIRT1 inhibition increases the expression of Bax, thereby promoting apoptosis. Activation of caspase‐3 results in reduced Bcl‐2 expression, which causes myocardial cell death by hypoxia/reperfusion.^15^ SIRT1 activators exert anti‐inflammatory effects via NF‐κB during myocardial I/R injury,^16^ and SIRT1/nuclear erythroid‐related factor 2 (NRF2) plays a central role in OS and stress‐induced apoptosis.^17^, ^18^ Interestingly, according to recent studies, SIRT1 levels in elderly rats are significantly increased by exercise, and the downstream target Pgc‐1α is also activated,^19^ thus reducing the probability of potentially fatal myocardial fibrosis and improving ventricular systolic function.^20^

Although studies have reported that SIRT1 is involved in apoptosis, inflammation, and OS in animal models of I/R, the involvement of the SIRT1 signaling axis in syringin‐mediated protection against I/R damage, either in rat or cell models of I/R, remains unknown. Therefore, the aim of this study was to characterize this potential interplay between syringin and SIRT1 signaling in the above models.

## 2 MATERIALS AND METHODS

### 2.1 Animals

Fifty‐six male Sprague‐Dawley rats (~70 days old, 200 ± 30 g) were obtained from Beijing VitalRiver Laboratory Animal Co. Ltd. All animal experiments were conducted according to the Guidelines for Care and Use of Laboratory Animals published by the National Institutes of Health of China, with the approval of the Animal Ethics Committee of The Third Affiliated Hospital of Qiqihar Medical University (approval number: 2021JSK22). Rats were maintained in a room with filtered air at 25°C, a 12/12 h light/dark cycle, and controlled relative humidity (55%). Standard food and water were provided ad libitum.

### 2.2 Experimental groups

Thirty healthy rats were randomly divided into three groups: (1) a sham operation group (sham group), (2) myocardial I/R injury operation group (I/R group), and (3) syringin treatment group (I/R + syringin group).

### 2.3 Myocardial I/R modeling

We established an I/R rat model based on previous studies,^21^ with modifications. Briefly, the rats were weighed. A small animal ventilator was turned on, and the heart was exposed. We used 4‐0 silk thread to ligate the anterior descending branch of the left coronary artery for approximately 30 min to induce ischemia. The threads were then loosened, and reperfusion was conducted for approximately 2 h. The animal was then anesthetized with 25% ethyl carbamate (5 ml/kg; 57399‐97‐0; Sigma). Through lead II of the ECG, we observed a marked elevation in the S‐T segment, which indicated the successful completion of ischemia. In at least half of the elevated S‐T segments, a subsequent drop was observed, and the T‐wave recovery indicated that reperfusion was complete. Rats in the syringin treatment group received 50 mg/kg syringin (HY‐N0824; MedChemExpress) intraperitoneally (i.p.) for 1 week, in accordance with previous studies.^7^, ^8^, ^22^ After the ultrasonic measurement, mice of each group were killed by cervical dislocation. The heart was taken out immediately, and myocardium from part of the MI area was collected and frozen for quantitative PCR (qPCR) and western blot (WB) analysis. The remaining myocardium was cut into 3 μm serial sections and used for Masson and terminal deoxynucleotidyl transferase‐mediated dUTP‐biotin nick end labeling (TUNEL) staining.

### 2.4 Infarction size measurement

We used TTC (T8877; Sigma) staining to determine infarction size 2 h after reperfusion. Briefly, the rats were euthanized, and the hearts were fixed in paraformaldehyde overnight and embedded in paraffin, after which they were cut into five 1‐ml‐thick slices. The paraffin was then removed from the tissues, which were cut into 4‐μm‐thick sections. Next, the cells were stained with TTC to determine the infarction volume. They were then placed on a light table and photographed on two sides. Different areas were specified. Infarction size was calculated by taking the infarction area volume as a percentage of the total left ventricle (LV) volume.

#### 2.4.1 ELISA

Interleukin (IL)‐6, IL‐1β, and tumor necrosis factor (TNF)‐α levels in the homogenized myocardial tissue and cell supernatant were measured with ELISA kits (MLB00C, D6050, and DTA00D; R&D Systems) according to the manufacturer's instructions. Briefly, the tissue homogenate was processed by centrifugation at 1000×*g* for 10 min at 4°C. The supernatants were then cultured with the ELISA reagent. Finally, the optical density (OD) was measured using a microplate reader (Multiskan MK33; Thermolab Systems).

### 2.5 TUNEL staining

A TUNEL staining analysis kit was used according to the manufacturer's instructions. Briefly, samples were treated with 3% H_2_O_2_ and then digested with proteinase K (20 µg/ml) at 25°C. After approximately 10 min, the samples were cultured with the labeled buffer (1:18) at 37°C. After approximately 2 h, the samples were incubated with biotinylated anti‐digoxin antibody (1:100) for about 30 min. Streptavidin‐biotin peroxidase was used to detect luciferin. Next, 3,3'‐diaminobenzidine (DAB) was used to dye the samples. The 3'‐OH terminal DNA fragment was labeled using terminal deoxynucleotidyl transferase with digoxigenin‐11‐deoxyuridine triphosphate (DIG dUTP) to measure cell death. Dead cells were stained brown, whereas healthy cells were colored. The death index refers to the ratio of brown‐stained nuclei to total nuclei. For each sample, nuclei from 10 regions were analyzed.

### 2.6 Cell culturing and H_2_O_2_ induction

H9c2 myoblast (ATCC) were cultured in a humidified incubator (5% carbon dioxide, 95% atmosphere, 37°C) containing 10% FBS, 1% streptomycin/penicillin, and high‐glucose DMEM. To induce I/R damage, H9c2 cells were challenged with 200 μM H_2_O_2_ for 6 h.

### 2.7 Reactive oxygen species (ROS) measurement

The fluorescent probe dichloro‐dihydro‐fluorescein diacetate (DCFDA; D399; Invitrogen™) was used to measure ROS generation. The cells were cultured with 10 μl DCFDA for 30 min at 37°C in darkness. We used a microplate reader (Infinite M200; Tecan) to measure the fluorescence intensity (Ex 488 nm/Em 525 nm).

### 2.8 Measurement of antioxidative enzyme levels

Cardiac tissues (50 mg) were dissected, carefully trimmed, weighed, and homogenized in PBS buffer (50 mm, acid/alkali: 7.5). After centrifugation at 1500×*g* for 10 min at 4°C, the supernatant was removed and placed on an ice plate. A BCA protein assay kit (P0012; Beyotime Institute of Biotechnology) was used to determine the protein concentration. Kits provided by the Beyotime Institute of Biotechnology were used to measure the levels (U/mg) of the antioxidative enzymes superoxide dismutase (SOD) (S0101S) and catalase (S0051).

### 2.9 qPCR

TRIzol® reagents (15596026; Invitrogen; Thermo Fisher Scientific, Inc.) were used for whole RNA extraction from H9c2 cells. RNA was reverse‐transcribed using a cDNA reverse transcription kit (R211; Vazyme; Biotech Co., Ltd.) at 42°C for 1 h and 75°C for 5 min. The SYBR™ Green PCR Master kit (Q111; Vazyme Biotech Co., Ltd.) was used for qPCR. Thermal cycling protocols were as follows: initial denaturation (95°C, 3 min); 40 cycles (95°C, 30 s; 56°C, 30 s; 72°C, 30 s). We used the 2(−ΔΔC(T)) method to calculate relative changes in gene expression,^18^ with GAPDH as an internal control for normalization of mRNA expression levels. All experiments were conducted in triplicate.

### 2.10 WB

RIPA lysis buffer was used for total protein extraction according to the manufacturer's instructions (89900; Thermo Fisher Scientific). We used a BCA protein analysis kit to measure the total protein concentration in the supernatant (P0012; Beyotime Institute of Biotechnology). Proteins were subjected to sodium dodecyl sulfate‐polyacrylamide gel electrophoresis and transferred onto a nitrocellulose membrane (Hybond‐C; Amersham Biosciences). The membrane was cultured first with primary antibody overnight and then with horseradish peroxidase‐conjugated secondary antibody. Band intensity was determined using BandScan 5.0 software.

### 2.11 Flow cytometry (FC) analysis

Transfected cells (1 × 10^6^) were centrifuged for 5 min at low temperature and high velocity (4°C, 100×*g*) and suspended in 200 µl of binding buffer with 10 µl annexin V‐FITC. Cells were resuspended in 5 µl PI + 300 µl PBS after centrifugation and then cultured for 30 min in darkness at room temperature. Cells were then stained using the Annexin V/PI Apoptosis Detection Kit (556547; BD Biosciences) according to the manufacturer's instructions. BD FACSCalibur™ FC (Becton; Dickinson and Company) was used to assess fluorescence. FlowJo software (version 7.6.1; FlowJo LLC) was used to analyze FC data.

### 2.12 Cell viability

We assessed cell activity using the Cell Counting Kit‐8 (CCK‐8) assay kit (ab228554; Abcam) according to the manufacturer's instructions. Briefly, the cells were seeded in a 96‐well plate, and 10 µl of CCK‐8 reagent was added to every well. The cells were then cultured for 2 h at 37°C. The OD at 150 nm was measured with a microplate reader (Infinite M200; Tecan).

### 2.13 Statistics

The results are expressed as the mean ± standard deviation. Differences among multiple groups and between two groups were analyzed using analysis of variance test with Tukey's post hoc test and Student's *t*‐test (GraphPad Prism 5.0), respectively. Differences with a *p*‐value less than 0.05 were regarded as significant.

## 3 RESULTS

### 3.1 Effect of syringin on H9c2 cell damage induced by H_2_O_2_ stimulation

**Figure 1:**
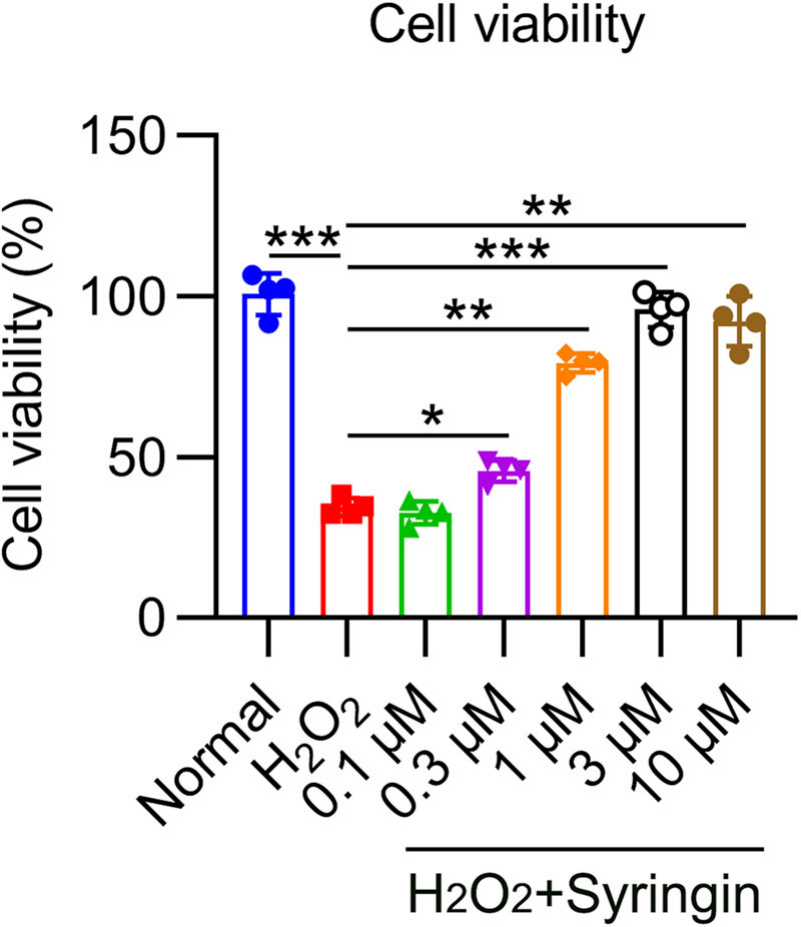
Effect of syringin administration of different concentrations in H_2_O_2_‐treated H9c2 cells. H9c2 cells were challenged with 200 μM H_2_O_2_ for 6 h, followed by coconditioning with syringin at the indicated concentrations. Cell viability was assessed by CCK‐8 assay. *p < .05, **p < .01, ***p < .001. CCK‐8, cell counting kit‐8.

To evaluate the in vitro effect of syringin on myocardial I/R damage, we established a cell‐based model in which I/R injury was induced by H_2_O_2_ treatment. The H_2_O_2_‐treated H9c2 cells were then treated with syringin at multiple dosages. The CCK‐8 assay was used to detect cell activity after H_2_O_2_ treatment. H_2_O_2_ treatment reduced cell viability to one‐third of the normal levels. Cell viability was significantly elevated by high concentrations of syringin (1, 3, and 10 μM), while a low concentration induced no significant changes (Figure 1).

### 3.2 Role of syringin in SIRT1 activation in H_2_O_2_‐treated H9c2 cells

**Figure 2:**
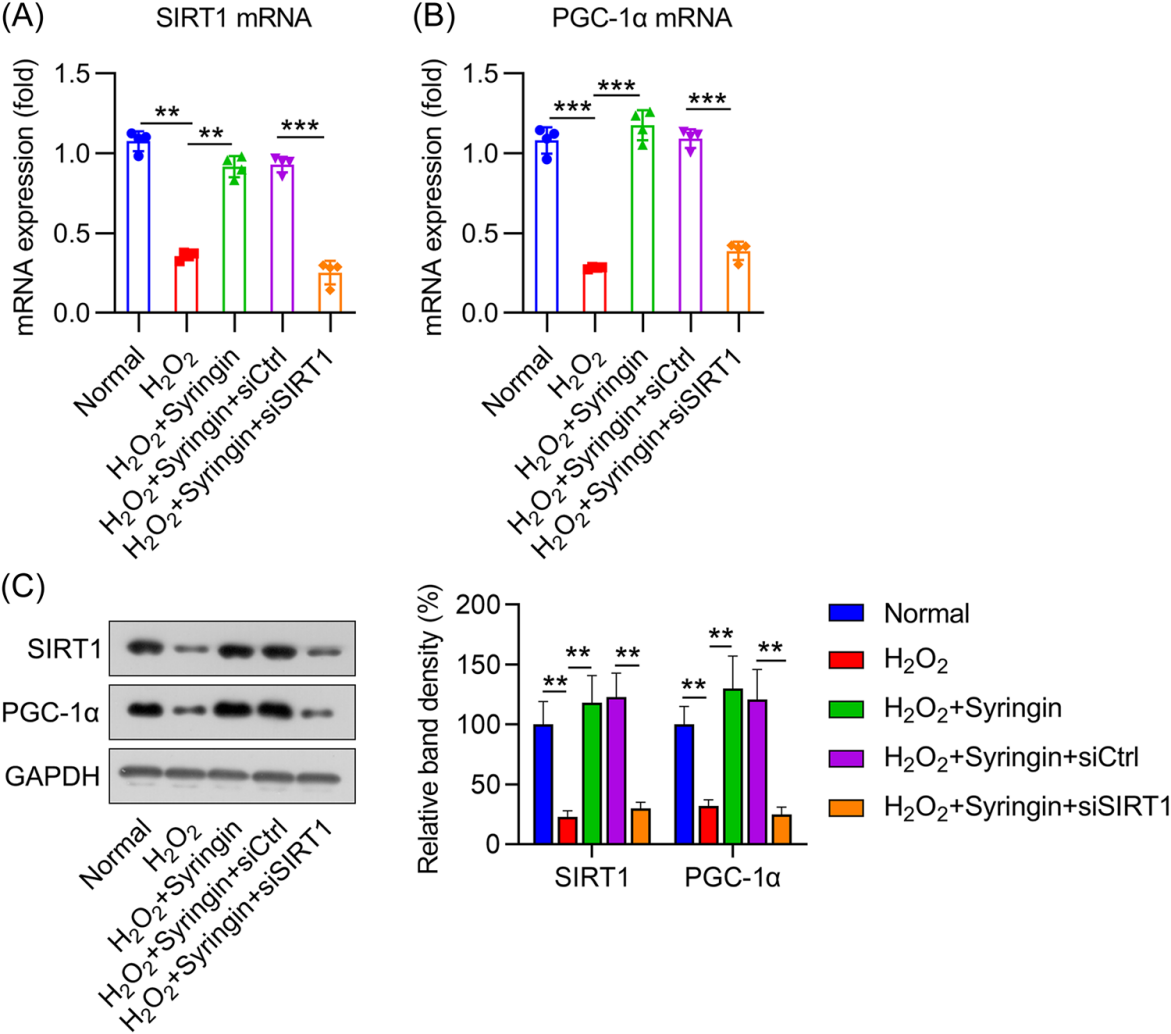
Effect of syringin administration on SIRT1 and PGC‐1α expression and confirmation of SIRT1 silencing in H_2_O_2_‐treated H9c2 cells. H9c2 cells were transfected with shRNA‐control (siCtrl) or shRNA‐SIRT1 (siSIRT1) for 12 h and then challenged with 200 μM H_2_O_2_ for 6 h, followed by coconditioning with 3 μM syringin. (A, B) qPCR data show the mRNA expression levels of SIRT1 and PGC‐1α in different groups of H9c2 cells. (C) Western blot indicates the protein expression levels of SIRT1 and PGC‐1α in different groups of H9c2 cells. **p < .01, ***p < .001. PGC‐1α, peroxisome proliferator‐activated receptor gamma coactivator 1 alpha; qPCR, quantitative PCR; shRNA, short hairpin RNA; SIRT1, sirtuin 1.

To analyze how syringin contributes to SIRT1 activation in cells treated with H_2_O_2_, we examined SIRT1 and PGC‐1α expression using qPCR and WB. qPCR data showed that the mRNA expression of both genes was reduced in H9c2 cells after H_2_O_2_ treatment; however, subsequent treatment with 3 μM syringin upregulated SIRT1 and PGC‐1α mRNA expression (Figure 2A,B). Similarly, WB analysis also showed that syringin administration restored the expression levels of SIRT1 and PGC‐1α, which had been reduced by H_2_O_2_ treatment (Figure 2C). These data suggest that the SIRT1 signaling axis could be activated by syringin treatment.

To evaluate the involvement of SIRT1 signaling in the response of H9c2 cells treated with H_2_O_2_ to syringin, cells were transfected with shRNA‐SIRT1 12 h before H_2_O_2_ and syringin treatment. qPCR and WB analyses showed that shRNA‐SIRT1 transfection downregulated SIRT1 at both the mRNA and protein levels (Figure 2A–C).

### 3.3 SIRT1 participated in the syringin‐mediated cardioprotective effect in H_2_O_2_‐treated H9c2 cells

**Figure 3:**
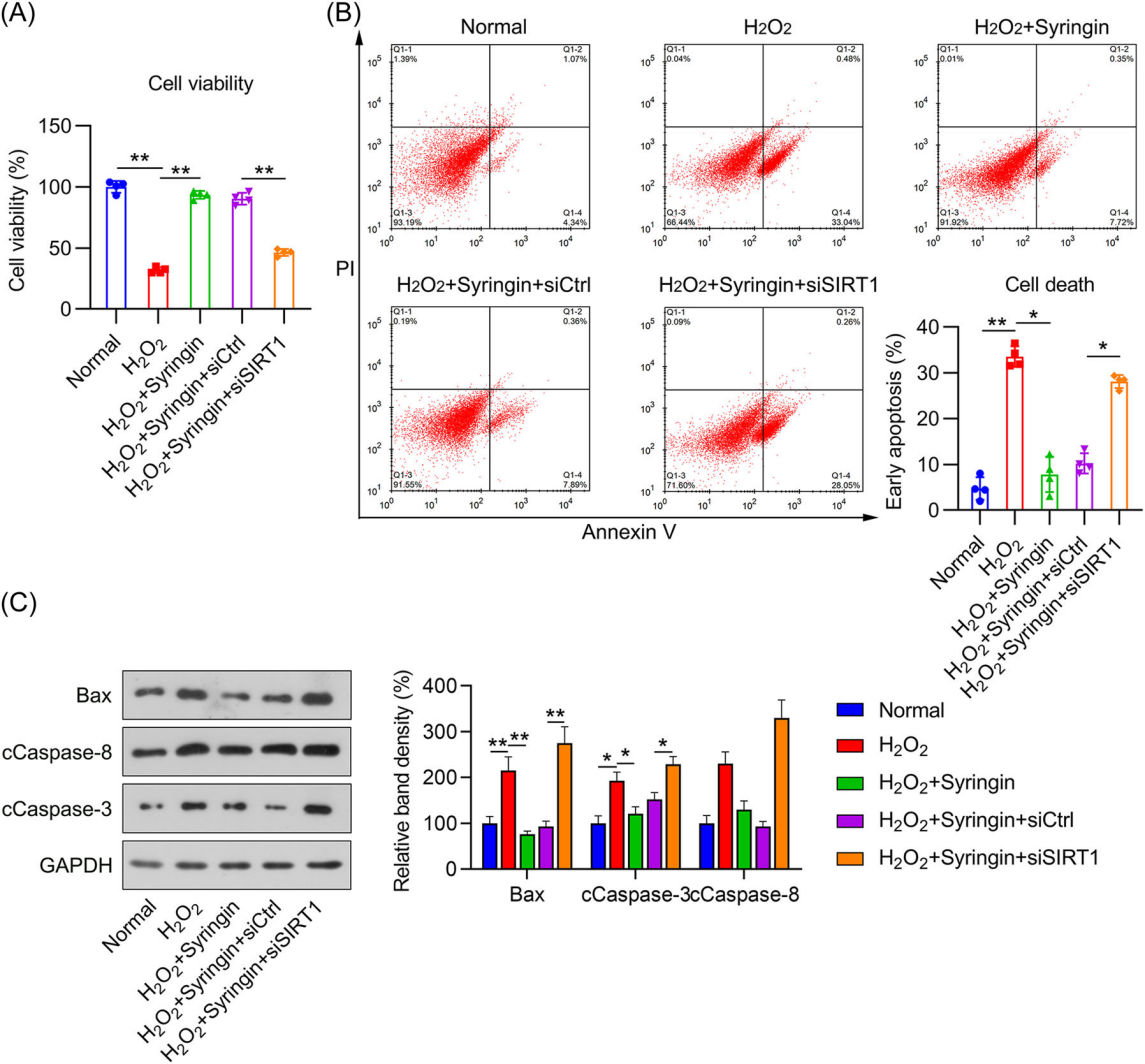
Effect of syringin and SIRT1 silencing on cell viability and apoptotic signaling in H_2_O_2_‐treated H9c2 cells. H9c2 cells were transfected with shRNA‐control (siCtrl) or shRNA‐SIRT1 (siSIRT1) for 12 h and then challenged with 200 μM H_2_O_2_ for 6 h, followed by coconditioning with 3 μM syringin. (A) The viability of H9c2 cells administered different treatments was assessed by CCK‐8 assay. (B) Flow cytometry with annexin V‐FITC and PI staining was conducted to examine the percentage of early apoptotic cells. (C) Protein levels of Bax, as well as cleaved caspase 3 and caspase 8, were examined by WB. *p < .05, **p < .01. CCK‐8, cell counting kit‐8; shRNA, short hairpin RNA; SIRT1, sirtuin 1; WB, western blot.

Next, we determined that SIRT1 conferred cardioprotective effects in H_2_O_2_‐treated H9c2 cells. Syringin exposure restored the viability of H_2_O_2_‐treated cells, but SIRT1 knockdown partially abolished the effect of syringin, causing impaired cell viability (Figure 3A). We also tested the effect of SIRT1 on the viability of both unstressed and injured cells. The results showed that SIRT1 deficiency reduced the viability of both types of cells, indicating that SIRT1 loss and restoration might be involved in the mechanism of H_2_O_2_‐induced cell damage and the protective function of syringin (Supporting Information: Figure 1).

FC with annexin V‐FITC/PI staining was then conducted to assess cell death. H_2_O_2_ exposure induced higher levels of H9c2 cell death than were observed in the control cells. Syringin treatment significantly reduced apoptosis in this I/R cell model. As expected, SIRT1 knockdown inhibited the syringin‐mediated prevention of cell death (Figure 3B). In addition, we examined apoptotic biomarkers, including Bax and cleaved caspase 3 and caspase 8, in H9c2 cells by WB. The results demonstrated that H_2_O_2_ stimulation induced increases in the protein levels of Bax, cleaved caspase 3 and caspase 8, but these levels were subsequently downregulated after treatment with syringin. Moreover, SIRT1 silencing was able to restore the H_2_O_2_‐induced upregulation in the protein levels of Bax and cleaved caspase 3 and caspase 8, even after syringin treatment (Figure 3C).

### 3.4 SIRT1 participated in syringin‐mediated inhibition of inflammation and OS in H_2_O_2_‐treated H9c2 cells

**Figure 4:**
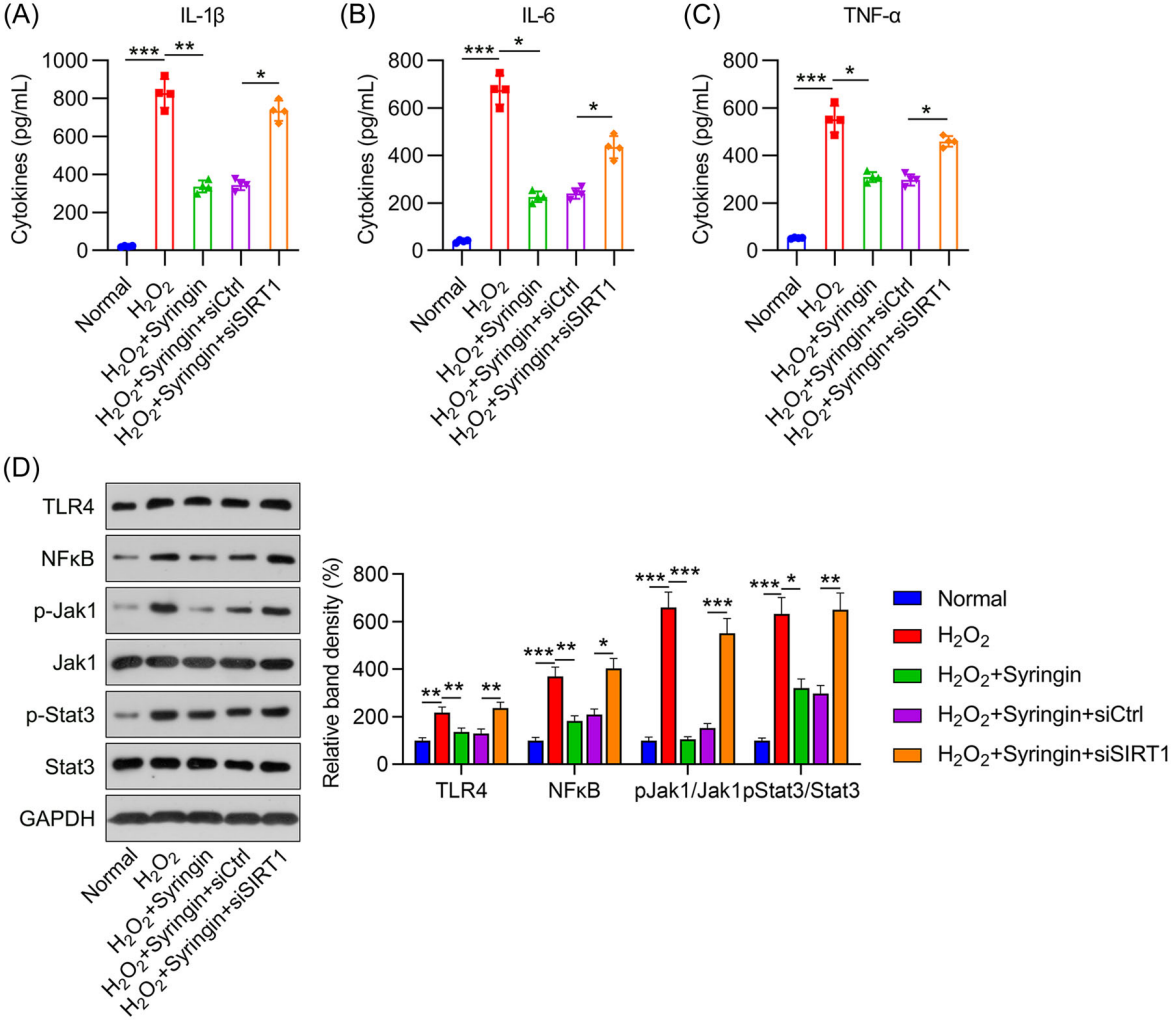
Effect of syringin and SIRT1 silencing on inflammation in H_2_O_2_‐treated H9c2 cells. H9c2 cells were transfected with shRNA‐control (siCtrl) or shRNA‐SIRT1 (siSIRT1) for 12 h and then challenged with 200 μM H_2_O_2_ for 6 h, followed by coconditioning with 3 μM syringin. ELISA was performed to detect the levels of released (A) IL‐1β, (B) IL‐6, and (C) TNF‐α. (D) Western blot indicates the protein expression levels of TLR4 and NF‐κB and the phosphorylation levels of Jak1 and Stat3. *p < .05, **p < .01, ***p < .001. shRNA, short hairpin RNA; SIRT1, sirtuin 1; TNF, tumor necrosis factor.

Inflammation and OS are two key characteristics of myocardial I/R; therefore, we examined inflammation and OS in H_2_O_2_‐treated H9c2 cells after treatment with syringin and knockdown of SIRT1. ELISA results indicated that the levels of proinflammatory cytokines (IL‐6, IL‐1β, and TNF‐α) were remarkably increased by H_2_O_2_ treatment. Conversely, syringin treatment significantly reduced cytokine levels compared to those in the control group. However, after SIRT1 knockdown, the inhibitory effect of syringin on inflammatory cytokine expression was abrogated (Figure 4A–C). In addition to SIRT1 signaling, we investigated the TLR4, NF‐κB, and Jak1/Stat3 pathways, which are all implicated in inflammation. Indeed, WB revealed that the levels of TLR4 and NF‐κB protein expression and of Jak1 and Stat3 phosphorylation were increased by H_2_O_2_ treatment, but subsequently reduced after syringin treatment. However, SIRT1 silencing abrogated the effect of syringin on TLR4 and NF‐κB protein expression and Jak1 and Stat3 phosphorylation (Figure 4D).

**Figure 5:**
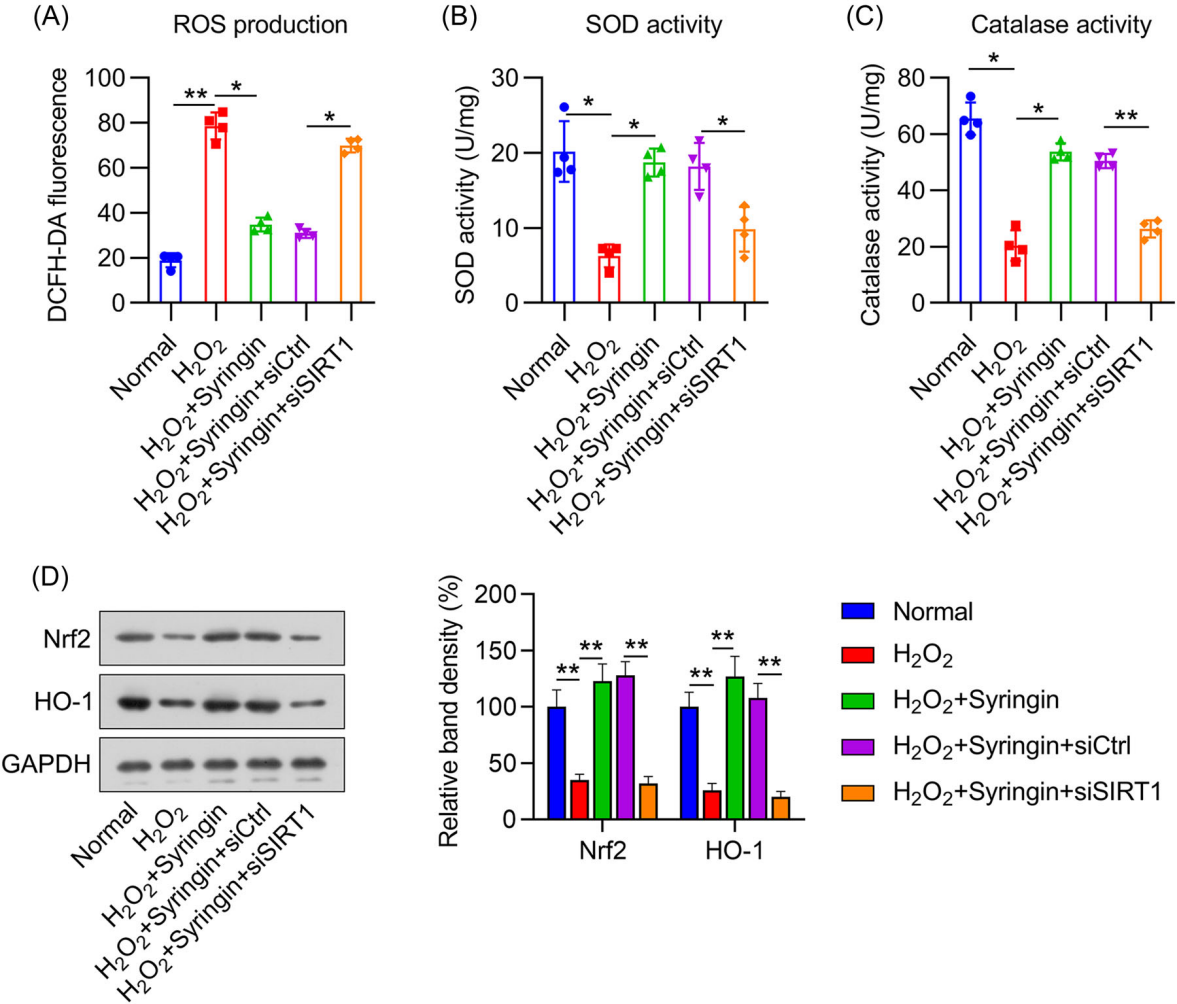
Effect of syringin and SIRT1 silencing on oxidative stress and the antioxidant response in H_2_O_2_‐treated H9c2 cells. H9c2 cells were transfected with shRNA‐control (siCtrl) or shRNA‐SIRT1 (siSIRT1) for 12 h and then challenged with 200 μM H_2_O_2_ for 6 h, followed by coconditioning with 3 μM syringin. (A) DCFH‐DA staining was performed to measure the ROS production. (B, C) Activity of the antioxidative enzymes SOD and catalase was determined using the corresponding assay kits. (D) Protein levels of NRF2 and HO‐1 were detected by western blot. *p < .05, **p < .01. NRF2, nuclear erythroid‐related factor 2; ROS, reactive oxygen species; shRNA, short hairpin RNA; SIRT1, sirtuin 1; SOD, superoxide dismutase.

The generation of ROS, activity of SOD and catalase, and activation of the antioxidative NRF2/heme oxygenase‐1 (HO‐1) pathway in H_2_O_2_‐treated cells were analyzed to evaluate the role of syringin in the antioxidant response. Indeed, H_2_O_2_ treatment produced ROS (Figure 5A), attenuated SOD and catalase activity (Figure 5B,C), and reduced NRF2/HO‐1 expression (Figure 5D). However, administration of syringin partially attenuated ROS levels, promoted SOD and catalase activity, and increased NRF2/HO‐1 expression. As before, SIRT1 silencing once again offset the effects of syringin (Figure 5A–D). These data clearly demonstrate that the effect of syringin on H_2_O_2_‐mediated inflammation and OS occurred in a SIRT1‐dependent manner.

### 3.5 Role of syringin in I/R injury and cardiac cell death in vivo

**Figure 6:**
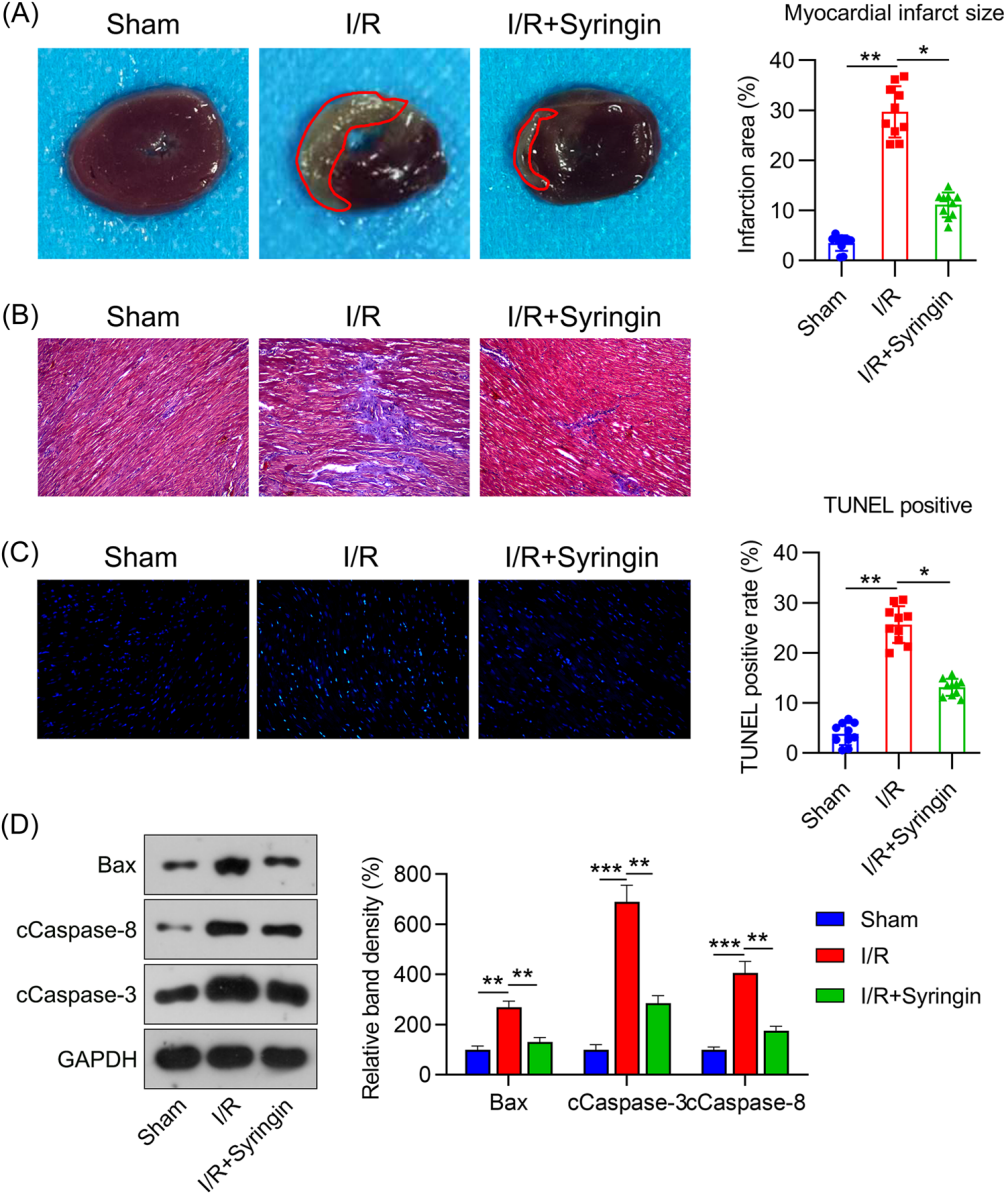
Syringin treatment protects against ischemia/reperfusion (I/R) injury in vivo. Syringin was administered intraperitoneally for 7 days in rats after I/R injury was induced. (A) Representative images of myocardial infarct in rats detected by triphenyltetrazolium chloride staining. The percentage of myocardial area affected by infarct is also shown. (B) Myocardial tissues of mice stained with Masson (×200). (C) Representative images of TUNEL staining and quantitative results. (D) Protein levels of Bax, as well as cleaved caspase 3 and caspase 8, were examined by WB. *p < .05, **p < .01. TUNEL, terminal deoxynucleotidyl transferase‐mediated dUTP‐biotin nick end labeling; WB, western blot.

To evaluate the cardioprotective influence of syringin in a rat model of myocardial I/R damage, syringin was administered i.p. for 7 days. TTC staining indicated a significant reduction in infarct size after syringin administration (Figure 6A). Likewise, Masson staining revealed that compared with the Sham group, the I/R group exhibited left ventricular dilatation, ventricular wall thinning, and obvious pathological myocardial fibrosis, but these pathological changes were ameliorated by syringin administration (Figure 6B). Furthermore, decreased levels of LVEF and LVFS were observed in the I/R injury model, while syringin treatment elevated these two parameters (Table 1).

**Table 1:** Syringin treatment improved cardiac function against I/R injury in vivo.

Parameters	Sham	I/R	I/R + syringin
LVEF (%)	62.3 ± 6.9	23.8 ± 5.2*	42.1 ± 5.6$
LVFS (%)	43.6 ± 3.9	19.7 ± 5.0*	31.3 ± 4.4$

*Note*: Cardiac function in rats was measured using ultrasound electrocardiogram.

Abbreviations: I/R, ischemia/reperfusion; LVEF, left ventricular ejection fraction; LVFS, left ventricular fractional shortening.

* *p* < .05 versus Sham group.

^$^ *p* < .05 versus I/R group.

The TUNEL assay was used to analyze the role of syringin in myocardial cell death. Representative images of TUNEL staining are shown in Figure 6C. The proportion of TUNEL‐positive cells was higher in the I/R group than in the sham group, and the proportion of TUNEL‐positive cells in the syringin group was remarkably lower than that in the I/R group (Figure 6C). Indeed, increased levels of Bax and cleaved caspase 3 and caspase 8 were observed in I/R rats. Conversely, syringin treatment reduced the level of these proteins (Figure 6D). These data suggest that syringin ameliorated myocardial injury and cell death caused by I/R.

### 3.6 Role of syringin in SIRT1 activation in myocardial tissue of rats with I/R

**Figure 7:**
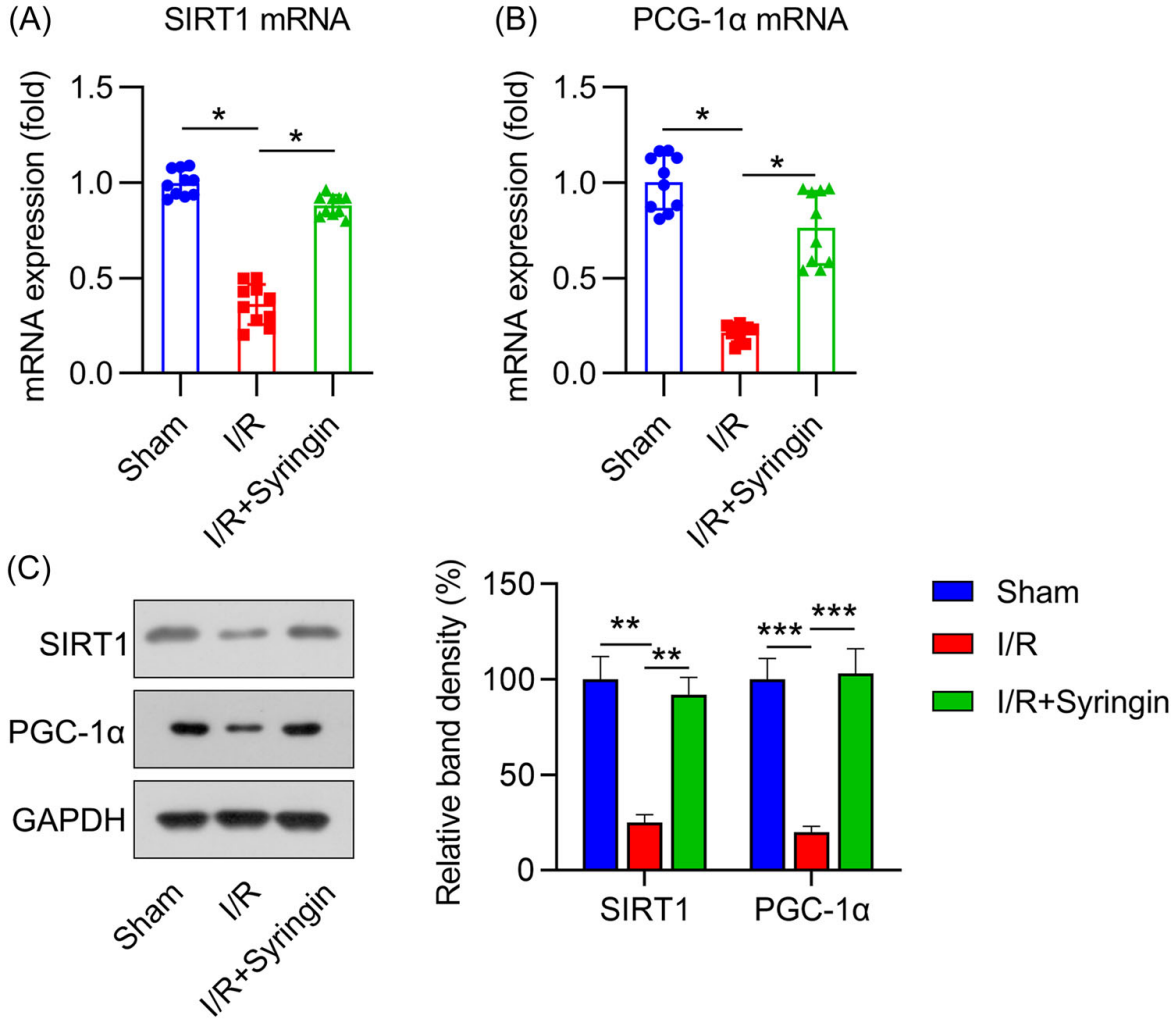
Syringin treatment upregulates myocardial Sirt1 and Pgc‐1α expression in ischemia/reperfusion (I/R) rats. Syringin was administered intraperitoneally for 7 days in rats after I/R injury was induced. (A, B) qPCR data show the mRNA expression levels of SIRT1 and Pgc‐1α in the homogenate of rat myocardial tissue. (C) Western blot shows the protein expression levels of SIRT1 and Pgc‐1α in the homogenate of rat myocardial tissue. *p < .05. PGC‐1α, peroxisome proliferator‐activated receptor gamma coactivator 1 alpha; qPCR, quantitative PCR; SIRT1, sirtuin 1.

Next, we assessed the role of syringin in SIRT1 activation in vivo. qPCR and WB showed that the I/R injury model exhibited SIRT1 and PGC−1α mRNA and protein downregulation in myocardial tissue (Figure 7A–C). Administration of syringin restored SIRT1 and PGC‐1α expression levels (Figure 7A–C).

### 3.7 Inflammation and antioxidative response in syringin‐treated I/R rats

To elucidate the mechanism of syringin‐mediated myocardial damage amelioration in I/R rats, proinflammatory cytokine levels and antioxidative responses were analyzed. First, ELISA data showed that the release of myocardial cytokines, including IL‐6, IL‐1β, and TNF‐α, was remarkably upregulated in the supernatant of cardiac tissue homogenates from the I/R group compared to that in the sham group.

**Figure 8:**
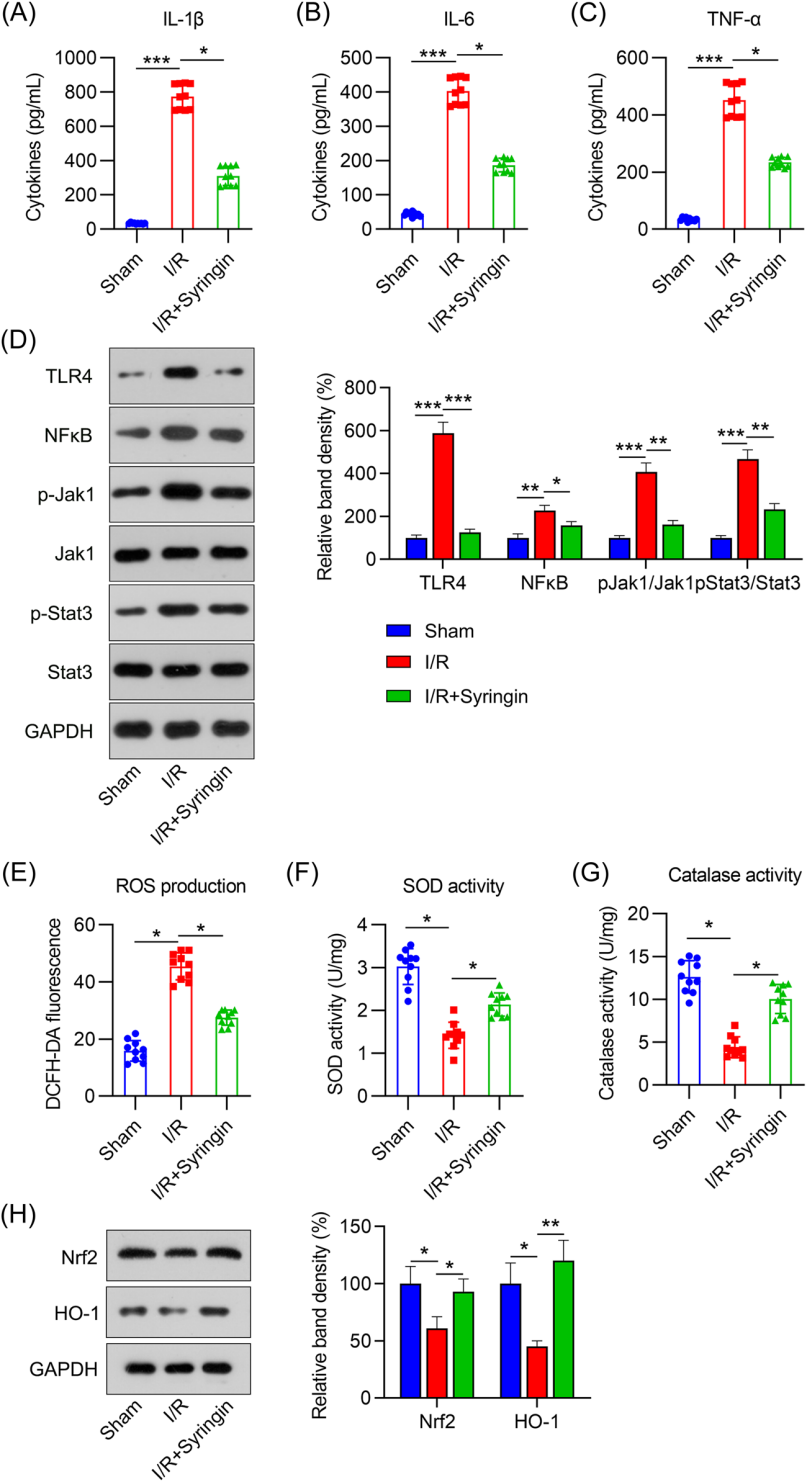
Syringin treatment attenuates myocardial inflammation and oxidative stress in ischemia/reperfusion (I/R) rats. Syringin was administered intraperitoneally for 7 days in rats after I/R was induced. ELISA was performed to detect (A) IL‐1β, (B) IL‐6, and (C) TNF‐α levels in rat myocardial tissue homogenate. (D) Western blot (WB) indicates the protein expression levels of TLR4 and NF‐κB and the phosphorylation levels of Jak1 and Stat3. (E) DCFH‐DA staining was performed to measure ROS production in rat myocardium. (F, G) Activity of the antioxidative enzymes SOD and catalase was determined using the corresponding assay kits. (H) Protein levels of myocardial NRF2 and HO‐1 in rats were detected by WB. *p < .05, ***p < .001. NRF2, nuclear erythroid‐related factor 2; ROS, reactive oxygen species; SOD, superoxide dismutase; TNF, tumor necrosis factor.

Subsequent treatment with syringin caused a significant reduction in cytokine levels in the myocardium (Figure 8A–C). Moreover, after I/R induction, levels of TLR4, NF‐κB, and phosphorylated Jak1 and Stat3 were increased, whereas this change was partially reversed after syringin administration (Figure 8D).

Likewise, ROS generation was increased in rats after I/R induction. Syringin administration significantly reduced ROS levels (Figure 8E). We next assessed the activity of two antioxidative enzymes, SOD and catalase. The data clearly indicated a significant reduction in enzyme activity following I/R induction. Syringin treatment partially restored enzymatic activity (Figure 8F,G). After I/R induction, NRF2/HO‐1 levels were reduced. However, after syringin administration, NRF2 and HO‐1 expression was partially restored (Figure 8H). These data clearly indicated that the myocardial inflammation and OS in the I/R model were alleviated by syringin administration.

## 4 DISCUSSION

**Figure 9:**
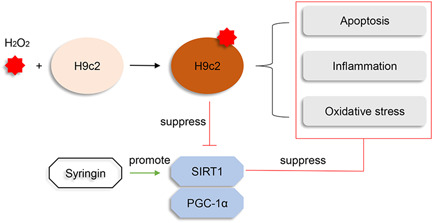
Schematic diagram for the mechanism of action of syringin on MI. Syringin protects heart function via the SIRT1/PGC‐1α signaling pathway in ischemia/reperfusion rats and H_2_O_2_‐treated H9c2 cells. Syringin reduces MI injury and ameliorates apoptosis, inflammation, and OS. These effects in vitro were abrogated through the silencing of SIRT1. MI, myocardial infarction; OS, oxidative stress; PGC‐1α, peroxisome proliferator‐activated receptor gamma coactivator 1 alpha; SIRT1/PGC‐1α, sirtuin 1/peroxisome proliferator‐activated receptor gamma coactivator 1 alpha.

MI has become the most common cause of death globally. Early reperfusion is currently the most common treatment approach, but even with this intervention the potential exists for severe myocardial damage, characterized by cytokine release, neutrophil infiltration, calcium overload, and ROS overproduction. Based on multiple laboratory studies and clinical trials, these effects remain unavoidable in many cases. Considering this, we explored a novel approach for ameliorating myocardial I/R injury by investigating whether syringin protects heart function via the SIRT1 signaling pathway in I/R rats and H_2_O_2_‐treated H9c2 cells. Our findings suggested that syringin reduced MI injury and ameliorated apoptosis, inflammation, and OS. These effects were eliminated in vitro by silencing SIRT1 (Figure 9), suggesting that the SIRT1 signaling pathway is involved in the cardioprotective effect of syringin.

To treat ischemic injuries, such as acute kidney injury, cerebral infarction, ischemic stroke, and MI, herbal medicine is often used.^23^, ^24^, ^25^ Several different drugs have been selected to prevent myocardial I/R injury.^9^, ^26^ In recent years, several studies have shown syringin to have a good regulatory effect on biological activity. For example, syringin injection decreases plasma glucose levels and increases plasma insulin levels in fasting Wistar rats by mediating the release of acetylcholine from nerve terminals, which has a positive effect on insulin secretion.^27^ Cui et al.^28^ demonstrated that eugenin effectively reduces sleep latency and increases sleep duration. Further research has revealed that NOS/NO pathway induction is the basis of these functions. Syringin also reduces NF‐κB activation, thereby reducing levels of inflammatory factors, which alleviates fulminant liver failure induced by lipopolysaccharide/d‐galactosamine administration.^29^ Liu et al.^8^ reported that syringin reduced cerebral I/R damage in rats, and that the neuroprotective functions of syringin could be related to the suppression of TLR4. Syringin also protects against intestinal inflammation by suppressing NF‐κB, while simultaneously activating the NRF2 signaling pathway in colitis.^30^

Our findings agree with previous research into the cardioprotection conferred by syringin against I/R injury. Both in vivo and in vitro studies have demonstrated that syringin administration results in reduced inflammation and OS, as evidenced by decreased inflammatory cytokine production and ROS levels, as well as increased antioxidant enzyme levels and associated signal transduction, including NRF2 signaling. Our in vitro experiment also showed that syringin exerted cytoprotective effects on H_2_O_2_‐treated H9c2 cells by preventing apoptosis. In vivo experiments clearly demonstrated that syringin could alleviate I/R‐induced MI and cell damage, as well as inflammation and OS. Collectively, our data strongly suggest that syringin confers a cardioprotective effect during myocardial I/R injury.

Previous studies have demonstrated that syringin regulated TLR4/NF‐κB and SIRT3/PGC1α pathways.^31^, ^32^ As SIRT1 is upstream regulator of both pathways,^33^, ^34^, ^35^ we hypothesized that syringin also could influence the expression of SIRT1. SIRT1 is expressed in all mammalian cell lines and was initially judged to be a nuclear protein.^36^ Indeed, the presence of SIRT1 in the nucleus is critical for cellular protection.^37^ All substrates of SIRT1 (such as proteins regulating chromatin folding, metabolism, and the stress response) are dysregulated by cardiac I/R injury.^38^ Conversely, SIRT1 induction protects the heart from I/R damage.^38^ In addition, SIRT1 overexpression results in less tubulointerstitial fibrosis and hypertrophy.^39^ In the heart, SIRT1 confers protection by increasing the levels of survival molecules (such as BCL‐XL and thioredoxin 1) and decreasing OS. Clinical studies have shown that resveratrol, a SIRT1 activator, has a benign effect on diastolic function in subjects with coronary heart disease.^40^ PGC‐1α, a substrate of SIRT1, controls cellular metabolic energy via both mitochondria‐dependent^41^ and mitochondria‐independent^42^ mechanisms. Previous investigations have shown that hydrogen sulfide,^9^ tallianine,^26^ and alpha‐lipoic acid^43^ all promoted SIRT1/Pgc‐1α signaling and reduced inflammation and OS in rat models of ischemia. In the present study, syringin administration remarkably increased SIRT1 expression, and likewise inhibited inflammation and OS in a SIRT1‐dependent manner.

## 5 CONCLUSION

In conclusion, the present study revealed that syringin, a bioactive compound isolated from *Eleutherococcus senticosus*, protects against myocardial I/R injury at a low dosage in vivo and in vitro. Specifically, its efficiency in repressing I/R damage, OS, inflammation, and cell death were demonstrated to be SIRT1‐dependent. These results confirmed that the SIRT1 contributes to syringin‐mediated protection against myocardial I/R in rats. Broadly, our findings provide both novel insight into the mechanism of cardioprotection conferred by syringin, as well as further evidence for its potential value in the development of new treatments for MI.

## AUTHOR CONTRIBUTIONS

Di Zhao and Haifeng Shao conceived the study and designed the experiments. Ketong Liu and Jian Wang contributed to the data collection, performed the data analysis, and interpreted the results. Di Zhao wrote the manuscript. Haifeng Shao contributed to the critical revision of article. All authors read and approved the final manuscript.

## CONFLICT OF INTEREST STATEMENT

The authors declarre no conflict of interest.

## Supporting information

Supplementary Figure 1

## Data Availability

The data that support the findings of this study are available from the corresponding author upon reasonable request.
