# Supplementary figures and images for: Syringin exerts anti‐inflammatory and antioxidant effects by regulating SIRT1 signaling in rat and cell models of acute myocardial infarction
Source: Immun Inflamm Dis. 2023 Feb 24;11(2):e775. doi: 10.1002/iid3.775 (PMC9950876; doi:10.1002/iid3.775)

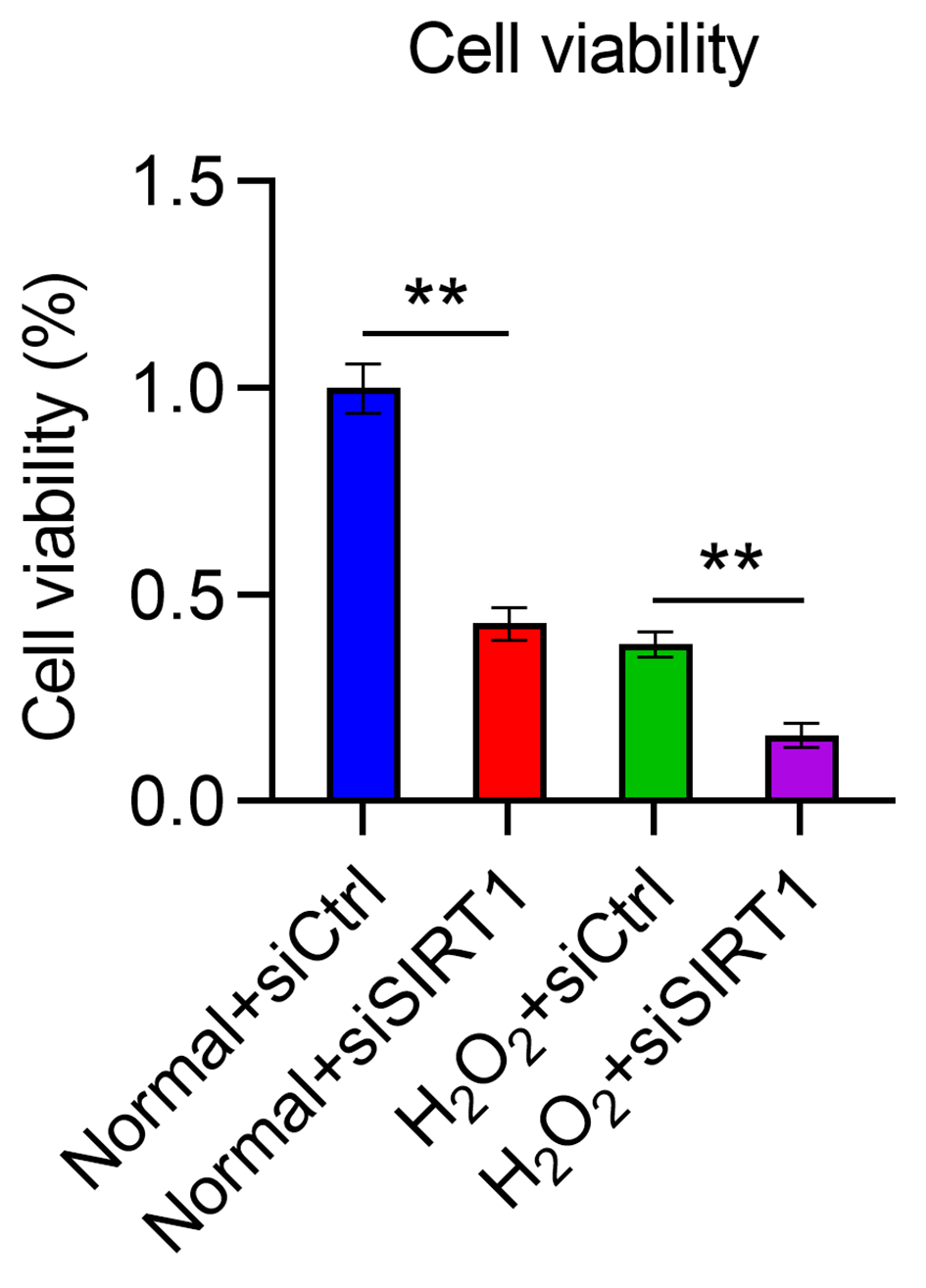

Supplement: Supplementary file 1 — Supplementary Figure 1 [file IID3-11-e775-s001.tif]
